# Mechanical Strain Regulates Osteogenic and Adipogenic Differentiation of Bone Marrow Mesenchymal Stem Cells

**DOI:** 10.1155/2015/873251

**Published:** 2015-04-02

**Authors:** Runguang Li, Liang Liang, Yonggang Dou, Zeping Huang, Huiting Mo, Yaning Wang, Bin Yu

**Affiliations:** ^1^Department of Orthopedics and Traumatology, Nanfang Hospital, Southern Medical University, Guangzhou 510515, China; ^2^Huiqiao Department, Nanfang Hospital, Southern Medical University, Guangzhou 510515, China; ^3^Key Laboratory of Bone and Cartilage Regenerative Medicine, Nanfang Hospital, Southern Medical University, Guangzhou 510515, China

## Abstract

This study examined the effects of mechanical strain on osteogenic and adipogenic differentiation of cultured MSCs by stimulating MSCs cultured in general and adipogenic differentiation media using a mechanical strain device. Markers of osteogenic (Runx2, Osx, and I-collagen) and adipogenic (PPAR*γ*-2, C/EBP*α*, and lipid droplets) differentiation were examined using real-time PCR, western blot, immunocytochemical, or histochemical stain analyses. Levels of Runx2 and Osx gradually increased in MSC groups in general medium subject to strain stimulation, as compared with in unstrained groups. After adding the stress signal, I-collagen protein levels of expression were obviously promoted in cells in comparison to the controls. The levels of PPAR*γ*-2 and C/EBP*α* were decreased, and the emergence of lipid droplets was delayed in MSCs groups in adipogenic differentiation medium subject to strain stimulation, as compared with in unstrained groups. Mechanical strain can promote differentiation of MSCs into osteoblasts and can impede differentiation into adipocytes. These results clarify the mechanisms underlying the effects of exercise on bone repair and reconstruction and provide a more adequate scientific basis for the use of exercise therapy in the treatment of obesity and metabolic osteoporosis.

## 1. Introduction

Obesity and osteoporosis are considered to be current worldwide public health concerns with serious detrimental implications for human health [1, 2]. Obesity is characterized by mass growth of adipose tissue, resulting from hypertrophy and increasing numbers of adipocytes [3]. Osteoporosis is characterized by decreased bone mass and is often accompanied by an increase in adipose tissue in the bone marrow, indicating gradual replacement of bone marrow by adipose tissue [4]. In vitro experiments have shown that both adipocytes and osteoblasts originate from common progenitor cells, mesenchymal stem cells (MSCs), which are capable of multiple pathways of differentiation, including differentiation into osteoblasts, chondrocytes, myoblasts, adipocytes, and fibroblasts [5, 6]. However, regulation of the initial phase of differentiation is complex and subject to the influence of multiple factors, such as growth factors, cytokines, and hormones. It is commonly accepted that, to induce MSC differentiation towards a specific cell type, it is necessary to simultaneously inhibit differentiation towards other cell types [7–9]. Transgenic techniques have been used to introduce a TAZ promoter to control the differentiation of MSCs toward either osteoblast or lipoblast phenotypes [10]. Zhou et al. [11] showed interactions between the TGF-*β* and Wnt signaling pathways during stimulation of chondrogenesis and inhibition of adipogenesis.

During normal development, organisms are exposed to a variety of mechanical stimuli that promote and regulate tissue development [12–14]. For example, physiological loading is widely believed to be beneficial in maintaining skeletal integrity [15], and skeletal unloading, as for example, during conditions of space flight, being bedridden, and fracturing of a limb, results in increased bone resorption and decreased bone mineral density, bone formation, bone strength, and mineralization [16, 17]. Mechanical stimuli acting on fat tissue, such as occurring through stretching, rubbing, and pressure during gymnastic exercise, massage, and whole-body vibration, are believed to decrease or prevent obesity and osteoporosis [18, 19]. Moreover, other potential and as yet unidentified mechanisms may exist to control fat production and osteoporosis, for example, as related to the regulation of osteogenic and adipogenic differentiation by biologically induced mechanical strain; such mechanisms, if identified, could potentially improve conditions for bone formation and increase caloric consumption to achieve weight loss goals.

This study aimed to investigate the effect of mechanical strain on the regulation of osteogenic and adipogenic differentiation of MSCs. Markers of osteoblast differentiation (Runx2, Osx, and I-collagen) and adipogenic differentiation (PPAR*γ*-2, C/EBP*α*, and lipid droplets) were observed in MSCs stimulated by a mechanical strain device, cultured in both general and adipogenic differentiation media in vitro. Results of the study provide a scientific basis for the application of improved exercise therapy regimens in the treatment of metabolic obesity and osteoporosis.

## 2. Materials and Methods

### 2.1. MSC Cultures and Identification

Sprague-Dawley rats (male and female, 80–100 g) were used for the isolation of marrow-derived MSCs. This animal study was approved by the Animal Care and Use Committee of Nanfang Hospital, Southern Medical University (Guangzhou, China). The rats were euthanized via cervical dislocation under the guidelines of the Animal Care and Use Committee of Nanfang Hospital; appropriate steps were taken to ameliorate suffering, in accordance with the guidelines of the committee. The MSCs were isolated according to a previously described method [20]. Bone marrow was obtained by flushing the femurs and tibias of rats with general medium consisting of DMEM-LG medium (Gibco, OK, USA) supplemented with 10% defined fetal calf serum (Gibco), 100 U penicillin, and 100 *μ*g/mL streptomycin (North China Pharmaceutical Factory, China). Isolated cells were plated in 25 cm^2^ flasks and incubated at 37°C in a humidified atmosphere containing 5% CO_2_. After 24 h, nonadherent cells were removed and washed with PBS, and fresh general medium was added to allow for further growth. Culture media were changed every 2-3 days. When the cells reached 80%–90% confluence, they were washed with PBS, detached using 0.25% trypsin, and then subcultured in two new 25 cm^2^ flasks at a concentration of 1 × 10^4^ cells cm^−2^. The MSCs were then collected at the second or third generation and analyzed by flow cytometry for expressions of CD29, CD34, CD44, and CD45 (BD, NJ, USA). All experiments were performed with cells at passages 3–6.

### 2.2. Group and Parameter Settings

**Figure 1:**
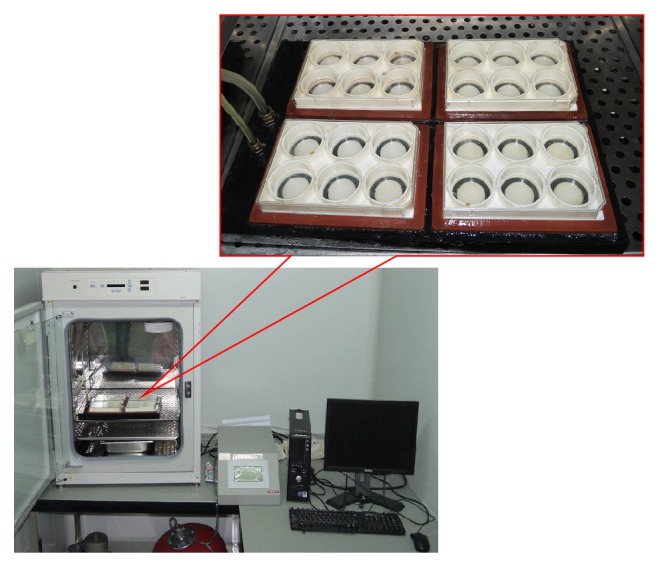
The Flexcell-5000 cell mechanical appliance was used. Cells were cultured in 6-well culture plates containing an elastic silicone membrane and fixed to the appliance module. A rubber gasket and silicone gel were applied to seal the gap between the modules and plates. A vacuum pump was connected to the module to generate negative pressure and induce periodical concave movement in the elastic membrane at the culture plate bottom. Thus, a force was applied along the surface of the radial tangent membrane to stimulate the cells attached to the membrane by the stretching stress. Tension was calculated by converting the stretching magnitude of the silicone membrane from its deformation rate (%).

Experimental and control groups were defined, respectively, by the presence or absence of stress intervention and intervention duration and by growth in an adipogenic differentiation or general medium/osteogenic medium. The strain parameters were as follows: 5% strain magnitude, 6 h/day, 10 times/min according to a sinusoidal waveform. The general medium was DMEM-LG medium, while the osteogenic medium consisted of 10^-8 ^mol/L dexamethasone, 10 mmol/L*β*-glycerol phosphate, and 50 *μ*g/mL L-ascorbic acid 2-phosphate. The adipogenic differentiation medium comprised DMEM-DG medium containing 1 *μ*mol/L dexamethasone, 0.5 mmol/L 3-isobutyl-1-methylxanthine (IBMX), 10 mg/L insulin, and 100 mmol/L indomethacin. The MSCs were stimulated with a Flexcell-5000 device (Flexcell International, NC, USA) (Figure 1), using the method described by Turner et al. [21] and Lohberger et al. [22].

### 2.3. Application of Mechanical Strain

We mixed adequate amounts of MSCs (passages 3–6) to ensure a consistent source of cells in each group. The MSCs were plated in general medium in collagen-coated Bioflex 6-well plates (Flexcell International, NC, USA) at approximately 1.5 × 10^5^/2 mL. Cells reached 80% confluence approximately 3 days later. After replacing the culture medium, the experimental groups were stimulated using the Flexcell-5000 device. All conditions in the control group were identical to those in the experimental group, except that mechanical stress stimulation was applied in the experimental group. Stress stimulation treatments were repeated four times, after which cells were then collected from each group. The mRNA and protein expressions of osteogenic and adipogenic markers in the MSCs were detected by real-time fluorescence quantitative polymerase chain reaction (PCR) and western blot analysis, respectively. I-collagen protein levels of expression in cells were detected by immunocytochemical stain. Lipid droplets were visualized by oil red O staining.

### 2.4. Reverse Transcription and Real-Time Fluorescence Quantitative PCR

Total RNA was extracted from cells subjected to mechanical strain and control cells using Trizol reagent (Invitrogen, CA, USA), according to the manufacturer's instructions. The results were quantified using spectrophotometry (ShiMadzu, Japan). The cDNA was synthesized from total RNA using the Prime Script RT Reagent Kit (TaKaRa, Japan) according to the manufacturer's instructions. Real-time fluorescence quantitative PCR analyses were performed at least in triplicate using a CFX96 real-time PCR system (Bio-Rad, Hercules, CA, USA). The primer nucleotide sequences used in the study are listed in Table 1. The information of primers came from the National Center for Biotechnology Information (NCBI) website (http://www.ncbi.nlm.nih.gov/gene/). The primer sequences were designed using Primer 3.0. Reactions were performed in a final volume of 20 *μ*L containing 25 ng cDNA, 10 *μ*L SYBR Premix Ex Taq (2x), and 1 *μ*L each of the PCR forward and reverse primers (10 *μ*M). The cDNA was amplified at 95°C for 10 min, followed by 44 denaturation cycles at 95°C for 10 s and annealing and extension at 60°C for 20 s, and at 72°C for 30 s. After the cycles, extension was performed at 72°C for 10 min. Glyceraldehyde-3-phosphate dehydrogenase (GAPDH) mRNA was quantified as an endogenous control, and the target gene expressions in each sample were normalized to those of GAPDH. The specificity of PCR was determined using a dissociation curve analysis. The data were analyzed using Bio-Rad CFX Manager Software (version 1.6, Applied Biosystems) to determine relative quantitative gene expressions.

### 2.5. I-Col Immunocytochemical Analysis

For immunocytochemical staining, cells were rinsed using ice-cold phosphate-buffered saline (PBS) and then fixed in 4% paraformaldehyde at 4°C for 30 min and washed three times in PBS. The silicone membrane was cut into small pieces. According to the instructions of SP/DAB kit (Wuhan Boster Biological Technology, LTD., Wuhan, China), the cells were incubated for 20 min at room temperature in 0. 1% Triton-X100, blocked with 3% bovine serum albumin, and incubated overnight at 4°C with I-col antibody (Santa Cruz Biotechnology, CA, USA) at 1 : 100 dilutions. The cells were washed with PBS and exposure to 1 : 200 dilution of secondary antibody for 40 min at room temperature. DAB Substrate Kit was used according to the manufacturer's protocol. Images were observed under a microscope and subsequently photographed.

### 2.6. Western Blotting

Proteins were extracted from the collected cells and were measured and analyzed using a BCA protein assay kit (Boster, China), according to the manufacturer's instructions. Equal amounts of protein from each sample were separated using SDS-PAGE (10% gel). The separated proteins were transferred to a nitrocellulose membrane using an electroblotting apparatus (Bio-Rad, Hercules, CA, USA). The membrane was blocked in 5% nonfat milk/PBS-Tween 20 solution, followed by separate incubation with monoclonal antibodies specific for Runx2 (1 : 1000; Santa Cruz Biotechnology, CA, USA), Osterix (1 : 1000; Santa Cruz Biotechnology, CA, USA), PPAR*γ* (1 : 1000; Santa Cruz Biotechnology, CA, USA), adiponectin (1 : 1000; Santa Cruz Biotechnology, CA, USA), and C/EBP*α* (1 : 1000; Santa Cruz Biotechnology, CA, USA) at 4°C overnight. After incubating at room temperature for 1.5 h, membranes were incubated for 1 h at room temperature with horseradish-peroxidase-conjugated secondary antibody (goat anti-mouse IgG, Wuhan Boster Biological Technology, Wuhan, China) diluted to 1 : 5000 in 5% nonfat milk/PBS-Tween 20. The membrane was washed three times with 0. 01% PBS/Tween 20 for 10 min after each antibody application. The nitrocellulose membrane proteins were detected using the ECL Plus Detection System (Amersham, Germany), according to the manufacturer's instructions. Various protein band intensities were quantified using densitometry and ImageJ software (National Institutes of Health, USA).

### 2.7. Histochemical Staining

After fixation in 4% paraformaldehyde, cells were rinsed three times for 5 min in deionized water and stained using cytoplasmic triglyceride droplets with oil red O [23, 24].

### 2.8. Statistical Analysis

All data were expressed as means ± standard error of the mean (SEM). Single-factor analysis of variance (ANOVA) was performed using SPSS version 11.5 statistical software. Values of *P* < 0.05 were considered statistically significant.

## 3. Results

### 3.1. MSC Morphology and Identification

The primary MSCs adhered to the culture flask wall at 2–4 days after seeding and were fibroblast-like and spindle-shaped in morphology. Clustering and rapid cell proliferation were observed after the first medium replacement at days 3 or 4. After multiple medium replacements and PBS washing, hematopoietic cells were gradually eliminated. At days 12–14, cell confluence reached 80%. After passaging, the growth rate of the MSCs increased, and full confluence was reached after 7–10 days. The cells were slender and spindle-shaped and were spirally distributed until cell fusion. As consistent with the known phenotypic characteristics of MSCs [25, 26], the cells tested positive for CD29 (98.6%), CD34 (1.3%), CD44 (99.4%), and CD45 (1.6%). The MSCs proliferated rapidly and reached 80% confluence by 48–72 h in 6-well culture plates containing elastic silicone membranes. After mechanical strain was applied, the cells were orientated perpendicular to the axis of the strain.

### 3.2. Effects of Mechanical Strain on Osteogenic Differentiation of MSCs

**Figure 2:**
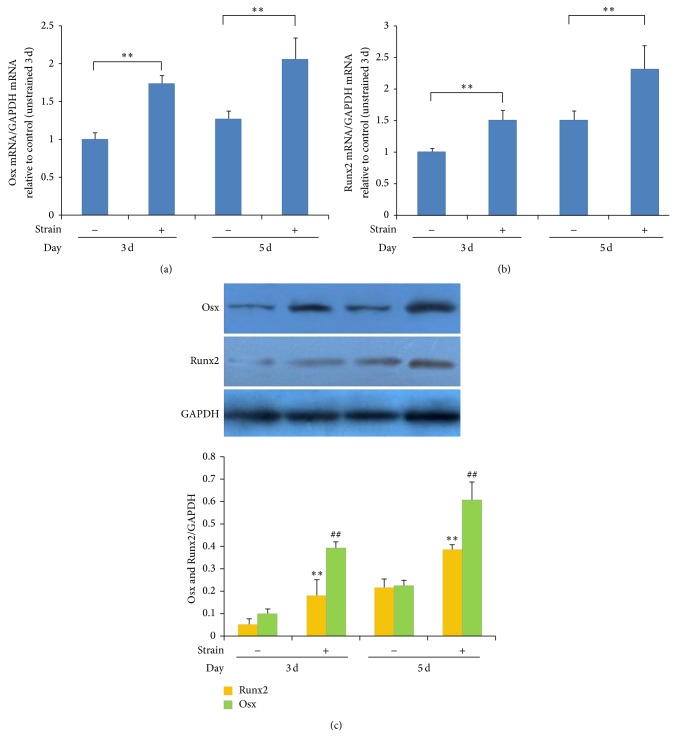
Mechanical strain promotes differentiation of MSCs into osteoblasts. Quantitative real-time PCR analysis showed that mRNA levels of Runx2 (a) and Osx (b) in general medium increased gradually in MSCs assigned to strain stimulation groups at 3 days and 5 days, as compared with levels in unstrained groups. The mRNA/GAPDH ratios in the different groups were measured by Bio-Rad CFX Manager Software. Values are expressed as means plus or minus standard errors of the mean (±SEMs) (*n* = 4); ^∗^ *P* < 0.05, ^∗∗^ *P* < 0.01: strain-stimulated groups were compared with corresponding unstrained control groups. (c) Western blot analysis showing that levels of Runx2 and Osx proteins gradually increased in the MSCs assigned to the strain stimulation groups at both 3 days and 5 days, as compared with unstrained groups. The mRNA/GAPDH ratios in the different groups were measured by the intensities of the protein bands, as quantified by densitometry with ImageJ software. Values are expressed as means ± SEMs (*n* = 4); ^∗^ *P* < 0.05, ^∗∗^ *P* < 0.01. Strain-stimulated groups were compared with corresponding unstrained control groups.

**Figure 3:**
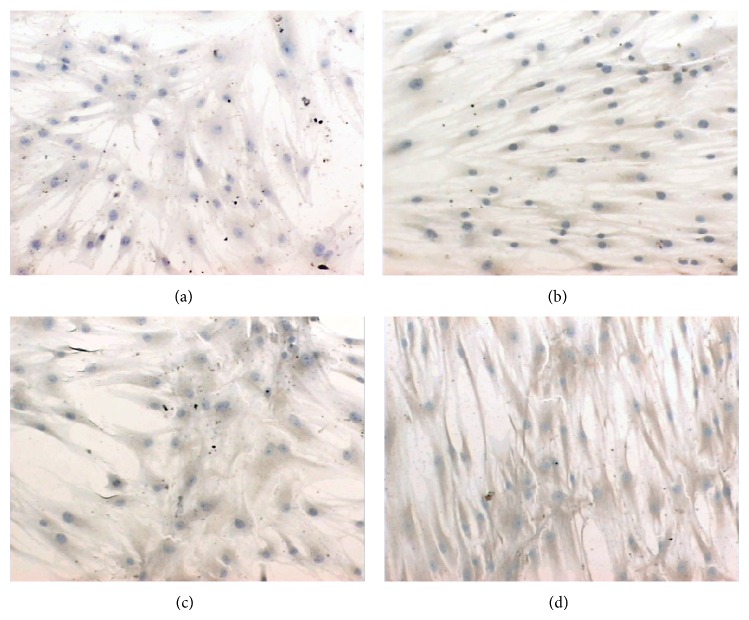
Mesenchymal stem cells (MSCs) under 7 days of unstrained in general medium (a), strain-stimulated in general medium (b), and unstrained in osteogenic medium (c), strain-stimulated in osteogenic medium (d). I-collagen protein levels of expression in cells were detected by immunocytochemical stain. I-collagen protein staining in the cytoplasm was stronger in the experimental groups (strain + osteogenic medium) at 7 days than in the control groups (general medium or osteogenic medium). Stressed cells were oriented perpendicularly to the strain axis, whereas, in the control group (unstrained), cells were randomly oriented.

Levels of Runx2 and Osx mRNA and protein gradually increased in MSCs in the strain stimulation groups in general medium at both 3 days and 5 days, as compared to unstrained groups (Figure 2). I-collagen protein staining in the cytoplasm was stronger in the experimental groups (strain + osteogenic medium) at 7 days than in the control groups (general medium or osteogenic medium). Stressed cells were oriented perpendicularly to the strain axis, whereas, in the control group (unstrained), cells were randomly oriented (Figure 3). These results show that mechanical stress can promote osteogenic differentiation of MSCs.

### 3.3. Effects of Mechanical Strain on Adipogenic Differentiation of MSCs

**Figure 4:**
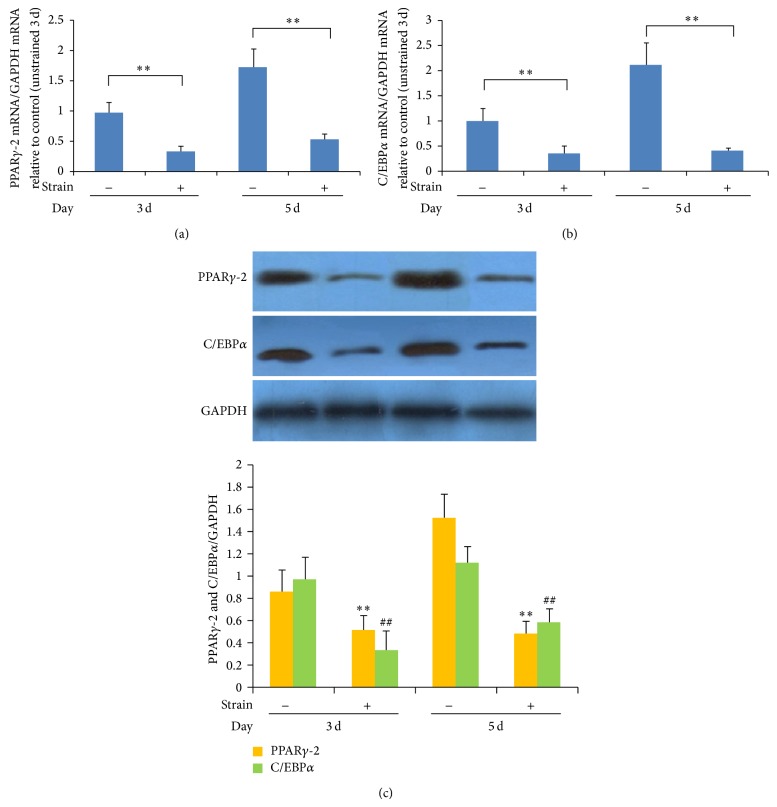
Mechanical strain impedes the differentiation of MSCs into adipocytes. Quantitative real-time PCR analysis shows that mRNA levels of PPAR*γ*-2 (a) and C/EBP*α* (b) decreased in MSCs assigned to strain stimulation groups in adipogenic differentiation medium at both 3 days and 5 days, as compared with levels in the unstrained groups. Levels of mRNA in the different groups were measured by Bio-Rad CFX Manager Software. Values are expressed as means plus or minus standard errors of the mean (±SEMs) (*n* = 3); ^∗^ *P* < 0.05, ^∗∗^ *P* < 0.01. Strain-stimulated groups were compared with corresponding unstrained control groups. (c) Western blot analysis showing that levels of PPAR*γ*-2 and C/EBP*α* proteins clearly decreased in MSCs assigned to strain stimulation groups in adipogenic differentiation medium at both 3 days and 5 days, as compared with levels in the unstrained groups. The mRNA/GAPDH ratios in the different groups were measured by the intensity of the various protein bands, as quantified by densitometry with ImageJ software. Values are expressed as the means ± SEMs (*n* = 3); ^∗^ *P* < 0.05, ^∗∗^ *P* < 0.01. Strain-stimulated groups were compared with corresponding unstrained control groups.

**Figure 5:**
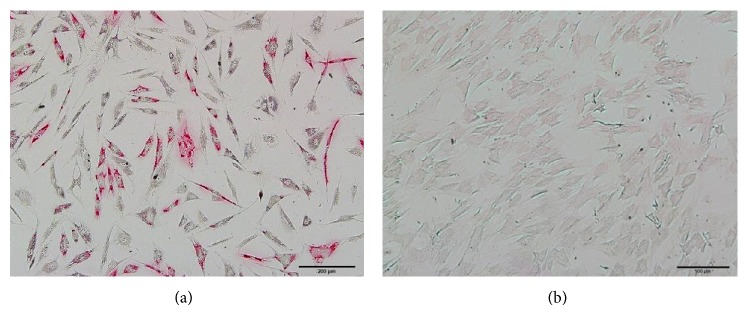
Mesenchymal stem cells (MSCs) under 10 days of unstrained (a) and strain-stimulated (b) conditions in adipogenic medium; the cells are stained with oil red O. (a) Numerous intracellular lipid droplets (colored red) formed in the unstrained group. (b) Few intracellular lipid droplets formed in the strain-simulated group.

Levels of PPAR*γ*-2 and C/EBP*α* mRNA and protein clearly decreased in MSCs assigned to strain stimulation groups in adipogenic differentiation medium at both 3 days and 5 days, as compared with levels in unstrained groups (Figure 4). After 10 days of sustained growth in adipogenic differentiation medium, lipid droplets appeared as translucent vacuoles with high refractive index inside the cytoplasm due to chemically induced differentiation to adipocytes. The presence of lipids was confirmed by oil red O staining of the droplets. In contrast, no significant development of intracellular lipid droplets was observed in the MSCs during the adipogenic differentiation process following the application of mechanical stress stimuli (Figure 5). These results show that mechanical stress can inhibit adipogenic differentiation of MSCs.

## 4. Discussion

Mechanical strain modulates a variety of cellular functions (such as proliferation and differentiation) that are critical for development, growth, and regeneration of various tissues in mammals [27]. Appropriate levels of strain are particularly important for regeneration and reconstruction of bone tissues after bone fracture [28, 29]. Obesity, a disease of excess adipose tissue, and osteoporosis, indicated by decreased bone mass, are both suppressed by exercise. These diseases can be further linked, as adipocytes and osteoblasts develop from common MSC progenitor cells [5, 30], and signals that promote MSC differentiation towards one lineage may preclude induction of the other, which shows relationships between diseases such as osteoporosis and obesity and their modulation by physical exercise [24].

Exercise can potentially reduce fat mass and increase bone mass through mechanical induction of MSCs away from adipogenesis and towards osteoblastogenesis. In our study, strain promoted MSC differentiation to osteoblasts. However, during lipoblast induction, stress stimulation delayed differentiation. Expressions of Runx2 and Osx were used as indicators of osteoblastic activity. The Runx2 protein is a transcription factor that is believed to act as a “master switch” that regulates a number of other genes involved in the development of bone-building cells (osteoblasts) [31]. The Osx protein contains a zinc finger domain located in the nucleus; thus, it is more specific than Runx2 for reflecting osteogenic processes. It is believed that Osx plays a role in osteogenic processes downstream of Runx2, indicating that it is involved in a late stage of osteogenic differentiation [32]. I-collagen protein is the most abundant extracellular protein in bone and is expressed during all stages of osteoblast development [33]. We found that I-collagen protein staining in the cytoplasm was stronger in the experimental groups (strain + osteogenic medium) at 7 days than in the control groups (general medium or osteogenic medium). In the study of Simmons et al. [34], application of equibiaxial cyclic strain to hMSCs cultured in osteogenic media stimulated a 2.3-fold increase in matrix mineralization over unstrained cells. So it was concluded that mechanical strain and osteogenic media had synergistic effects on osteogenic differentiation of MSCs.

The PPAR*γ* and C/EBP*α* proteins are critical transcription factors in the adipogenic differentiation of MSCs and are therefore known to play key roles in adipogenic processes [35, 36]. Expression of these transcription factors alone is sufficient to initiate adipogenic differentiation; adipocytes are completely absent in C/EBP*α*-knockout mice [37], while PPAR*γ* knockout mice show seriously flawed development of adipocytes [38]. Case et al. [39] reported that mechanical strain inhibits rosiglitazone-induced adipogenesis in bone marrow-derived MSCs, a process that involves limiting the expression of PPAR*γ*-2. Intracellular lipid droplet formation is a sign of adipogenic differentiation and maturity of MSCs, although it appears relatively late in the adipogenic process [21]. The MSCs cultured in general medium and without stimulation showed weak adipogenic differentiation ability. It is difficult to observe the inhibitory effects of mechanical stress on adipogenic differentiation of MSCs using pure stimulation; therefore, in this study, we applied a composite stress stimulation comprising chemically induced adipogenic differentiation as well as mechanical strain, using the method described by Turner et al. [21]. The effect of stress on adipogenic differentiation of MSCs can be observed through enhanced adipogenic differentiation.

In this study, the mRNA and protein levels of intracellular Runx2 and Osx significantly increased at 3 days and 5 days in MSCs assigned to mechanical strain stimulation groups, as compared with those of the control group, while the expressions of PPAR*γ*-2 and C/EBP*α* were significantly reduced at 3 days and 5 days. Intracellular lipid droplets were observed in some of the MSCs after 10 days in the chemically induced adipogenic differentiation group, while no significant intracellular lipid droplets were observed in the mechanically strained group. These results suggest that appropriate mechanical stress stimulation can promote osteogenic differentiation, while impeding adipogenic differentiation. A number of studies have linked the upregulation of osteogenic genes in MSCs (such as Runx2, Osx, collagen type I, BMP-2, ALP, and osteocalcin) following mechanical stimulation [22, 40, 41]. However, differentiation of MSCs to osteoblasts occurred only under a certain range of stress stimulation magnitudes: when the stress was too high, biological activity of MSCs was inhibited, or generated cell damage or apoptosis [42]. We found that excessive magnitude of the cyclic tensile strain (>12%) induced oxygen free radical experimental groups according to the preliminary study disequilibrium, resulting in cytotoxicity [43].

In vitro, cell response to mechanical signals results in a variety of sensitive gene expressions. These include synthesis and secretion of cytokines, which regulate cell function activity networks and mediate cascading amplification effects. Mechanical strain activates specific functional gene expressions via positive or negative feedback effects and regulation at transcriptional and translational levels, which triggers a series of biological effects such as cell proliferation, differentiation, and migration [14, 44]. The specific mechanisms underlying the regulation of MSC differentiation by mechanical strain is not clear but might be related to the regulation of transcription through various pathways in response to mechanical stress signals [7, 45–47]. Kearney et al. [42] reported that strain regulates MSC differentiation leading to osteogenesis. They examined mechanotransduction, in which cells were strained in the presence of a stretch-activated cation channel (SACC) blocker, gadolinium chloride (GdCl3), extracellular signal-regulated kinase (ERK) inhibitor (U0126), p38 inhibitor (SB203580), and phosphatidylinositol 3-kinase (PI3-kinase) inhibitor (LY294002). Following exposure to strain, the osteogenic markers Cbfa1, collagen type I, osteocalcin, and BMP2 were temporally expressed. Exposure to strain in the presence of GdCl3 reduced induction of collagen I expression, thus confirming the role of SACCs, at least in part, as mechanosensors involved in strain-induced MSC differentiation. It was found that strain-induced synthesis of BMP2 was reduced by inhibitors of kinases, ERK, p38, and PI3 kinase. Charoenpanich et al. [40] found that cyclic tensile strain enhances the modulation of genes associated with increases in both the proliferation and osteogenic differentiation of human MSCs from osteoporotic donors; potential mechanisms of strain-induced osteogenesis include the canonical pathways that are known to play an important role in bone formation, such as Wnt/BMP/PIK signaling. Tanabe et al. [48, 49] showed that mechanical stress downregulated the expression of PPAR*γ*-2 in 3T3-L1 cells and impeded adipocyte fat cell maturation. They found that expression of C/EBP, which is a member of the transcription factor family and plays a role in the regulation of fat cells, was significantly decreased by mechanical stress and concluded that the effect is related to the ERK/MAPK signal pathway. Hossain et al. [50] reported that compressive forces could inhibit human adipogenesis by suppressing expression of PPAR*γ*-2 and C/EBP*α* in a COX-2-dependent manner. Khayat et al. [23] showed that relatively low-frequency mechanical stimulation (0.01 Hz) could inhibit adipogenic differentiation of C3H10T1/2 mouse MSCs, even in a potent adipogenic differentiation medium. Furthermore, ERK signaling was decreased in a mechanically stimulated culture, indicating its role in the inhibition of adipogenic differentiation. Zayzafoon et al. [51] found that microgravity (weightlessness) inhibits osteoblast differentiation of human MSCs but promotes adipocyte differentiation. They hypothesized that weightlessness leads to decreased RhoA activity, affecting the activation of downstream Rho kinases, and impedes the phosphorylation of LIM-kinase and cofilin, thereby influencing the differentiation fate of stem cells.

In conclusion, this study showed that an appropriate mechanical signal can promote osteogenic differentiation, while suppressing adipogenic differentiation of MSCs. These results indicate that exercise can promote bone growth and impede the differentiation and maturation of adipocytes. Thus, this study provides a more adequate scientific basis for exercise therapy in the treatment of metabolic obesity and osteoporosis, as well as furthering our understanding of tissue engineering involving MSCs.

## Figures and Tables

**Table 1:** Design of primer sequences.

Gene	Reference gene ID	Primer	Primer sequence
Osx	NM_001037632.1	Forward	GTTCACCTGTCTGCTCTGCT
		Reverse	TTGGCTTCTTCTTCCCCGAC

Runx2	NM_001278484.1	Forward	CAGTTCCTAACGGGCACCAT
		Reverse	TTAGGGTCTCGGAGGGAAGG

PPAR*γ*-2	NM_013124.3	Forward	TCTGGGAGATCCTCCTGTTG
		Reverse	CGAAGTTGGTGGGCCAGAAT

C/EBP*α*	NM_012524.2	Forward	GCCGGGAGAACTCTAACTCC
		Reverse	TCGATGTAGGCGCTGATGTC

GAPDH	NM_017008.3	Forward	TGCCACTCAGAAGACTGTGG
		Reverse	TTCAGCTCTGGGATGACCTT
